# Designs and Techniques That Improve the Pullout Strength of Pedicle Screws in Osteoporotic Vertebrae: Current Status

**DOI:** 10.1155/2014/748393

**Published:** 2014-03-03

**Authors:** Thomas M. Shea, Jake Laun, Sabrina A. Gonzalez-Blohm, James J. Doulgeris, William E. Lee, Kamran Aghayev, Frank D. Vrionis

**Affiliations:** ^1^H. Lee Moffitt Cancer Center & Research Institute, Neuro-Oncology Department, Tampa, FL 33612, USA; ^2^Department of Chemical & Biomedical Engineering, University of South Florida, Tampa, FL 33620, USA; ^3^Morsani College of Medicine, University of South Florida, Tampa, FL 33612, USA; ^4^Department of Mechanical Engineering, University of South Florida, Tampa, FL 33620, USA; ^5^Departments of Neurosurgery and Orthopedics, Morsani College of Medicine, University of South Florida, Tampa, FL 33612, USA

## Abstract

Osteoporosis is a medical condition affecting men and women of different age groups and populations. The compromised bone quality caused by this disease represents an important challenge when a surgical procedure (e.g., spinal fusion) is needed after failure of conservative treatments. Different pedicle screw designs and instrumentation techniques have been explored to enhance spinal device fixation in bone of compromised quality. These include alterations of screw thread design, optimization of pilot hole size for non-self-tapping screws, modification of the implant's trajectory, and bone cement augmentation. While the true benefits and limitations of any procedure may not be realized until they are observed in a clinical setting, axial pullout tests, due in large part to their reproducibility and ease of execution, are commonly used to estimate the device's effectiveness by quantifying the change in force required to remove the screw from the body. The objective of this investigation is to provide an overview of the different pedicle screw designs and the associated surgical techniques either currently utilized or proposed to improve pullout strength in osteoporotic patients. Mechanical comparisons as well as potential advantages and disadvantages of each consideration are provided herein.

## 1. Introduction

Osteoporosis is a disease condition broadly described as a generalized decrease in bone mass and associated decline in the architectural makeup of bone tissue, with a resultant decrease in bone strength and an increased risk in the incidence of bone fractures [1, 2]. This condition, affecting men and women of many different age groups and populations, is defined by The World Health Organization (WHO) as a bone mineral density (BMD) 2.5 standard deviations or more below the mean for healthy young white women, as cited by Kanis et al. [1]. Previous investigations have revealed that the risk for bone fractures increases proportionally by 1.5 to 3 times for each standard deviation reduction with respect to the healthy mean of BMD and that the lowest density is found at the site of a fracture [1, 3].

Osteoporosis has a great impact in spinal disorders and their treatment. Vertebral fractures are the most common type of osteoporotic fracture and responsible for 42–48% of the variation in kyphosis in patients with osteoporosis [4]. In osteoporotic patients with conditions such as kyphosis or compression fractures, complications originate with the continuous failure of conservative, nonsurgical treatments. This often leads to surgical intervention. However, a principal obstacle that is often encountered with surgical intervention in osteoporotic patients is the possibility of hardware pullout in spinal fusion due to the fragile characteristic of the bone, which can result from either micromotions/injuries or excess force applied at the bone-metal boundary [5]. Furthermore, by-product kyphosis, adjacent level kyphosis after instrumentation (known as junctional kyphosis), can occur in a patient with osteoporosis, which may alter the number of levels involved in the surgical intervention [5]. For these reasons, fusion devices used for osteoporotic patients require specific attention and design enhancement to improve the strength of the bone-screw interface. Thus, these fusion devices are of great interest to the research community.

The first screw intended for spinal surgery was likely utilized by Dr. King [6] in the mid-1940s, when he attempted to stabilize a lumbar spine by placing screws through the lateral articulations. In the late 1950s, Boucher [7] furthered Dr. King's idea by placing long screws through the pedicle which, a few decades later, led to the addition of a rod to simultaneously fuse multiple levels together [8]. This pedicle screw system, often referred to as the bilateral pedicle screw system (BPSS), has become the gold standard technique for spinal fusion.

Despite all the advancements in spinal fusion, achieving optimal pedicle screw fixation within bone of compromised quality is still a concern [9–14]. A variety of designs and surgical techniques have been implemented, both clinically and in laboratory settings, in an attempt to enhance the amount of fixation possible between the pedicle screw and the surrounding bone of osteoporotic patients. One method includes augmenting the screw with a bone cement, such as polymethylmethacrylate (PMMA), calcium phosphate (CaP), and hydroxyapatite (HA), which can dramatically increase the purchase of screws in the vertebral body [11, 13, 15–29]. Another approach for improving the screw fixation is design alteration, which includes modifications to the diameter [30–32], length [32, 33], thread design [34–38], and tapering of the inner diameter of the screw [17, 30, 38, 39]. Studies have shown that an increased diameter of the screw may increase fixation and stability; however, the screw diameter is limited by the anatomy of the pedicle and the risk of a pedicle fracture. Alternatively, expandable screws have been proposed to create an increase in diameter at either its distal end [12, 18, 21, 22, 40–44] or more medially just past the posterior cortex [10, 45] after its implantation, reducing the risk of pedicle fracture but increasing the purchase in the vertebral body.

As a surgical technique option, altering the screw trajectory from the traditional transpedicular approach to one that involves more of the screw threading being engaged with the cortical bone will, in turn, improve the anchoring of the screw [14]. Likewise, the addition of hooks/claws in the fusion procedure may increase the purchase and pullout strength of a spinal fusion [46, 47], especially at the ends of a construct.

Pullout strength testing under laboratory setup is generally the first step in evaluating the efficacy of a new spinal fusion technique/instrumentation that promises to enhance the purchase of an implant. For a pedicle screw, the pullout test consists of applying a gradual axial force at a constant rate of displacement to a screw that has been inserted into either the pedicle of a vertebra or within a block of “synthetic bone,” where the maximum force required before the screw loses its fixation within the spine is measured. This maximum force is referred to as the pullout force. While this type of axial failure may not be commonly seen in a clinical setting, its simplicity and reproducibility allow it to be considered as the most efficient method to compare a screw's anchorage within bone [48, 49].

This paper reviews different pedicle screw designs and implantation techniques that have been proposed for osteoporotic patients and have been evaluated under laboratory setup for pullout strength.

## 2. Basic Screw Design

**Figure 1:**
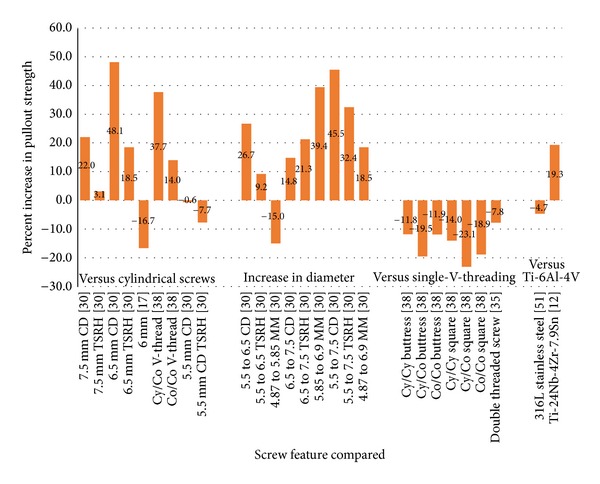
Effect of Screw Design on Pullout Strength. The percentages are with respect to a classic pedicle screw of otherwise similar dimensions TSRH: Texas Scottish Rite Hospital (conical screw), CD: Cotrel-Dubousset (conical screw), MM: Moss Miami (cylindrical screw), Cy/Cy: cylindrical thread with cylindrical core, Cy/Co: cylindrical thread with conical core, Co/Co: conical thread with conical core, V: standard thread, and Ti: titanium.

Much needs to be considered when determining the proper pedicle screw design to be used for spinal fusion in an osteoporotic patient. Increasing the diameter and length of the screw has the potential to produce larger pullout forces, but they also increase the risk of fracturing the surrounding, fragile bone [30–32]. Tapering the diameter of the screw is believed to help compress surrounding bone, which may in turn enhance the screw's fixation in the vertebra [9, 17, 30, 34, 38, 39]. Different thread designs serve a diverse range of mechanical functions that must take into consideration the material properties of the bone that it is to be paired with [34–36, 38, 50]. Moreover, a screw material that would offer not only excellent mechanical properties but also exceptional biocompatible properties is crucial for successful long term performance [12, 51]. Design alteration is a topic of interest in the literature and the pros and cons of altering each design characteristic are individually discussed.  Figure 1 demonstrates the relative pullout strength of the various screw designs.

### 2.1. Pedicle Screw Size

Quite possibly the most intuitive technique used to achieve a high amount of purchase, where little is expected, is to increase the diameter of the pedicle screw [30–32]. After all, it is believed that amongst the anatomical considerations of pedicle screw design, the size of the outer diameter best influences pullout strength [52]. Hsu et al. [30] showed that there was a steady increase in the pullout strength as the diameter increased by approximately 1 mm, when testing three different diameters. Zindrick et al. [32] observed significantly higher pullout forces in 6.5 mm cancellous screws than in either 4.5 mm cortical screws or 4.5 mm Louis screws. Likewise, Patel et al. [31] tested cancellous and cortical screws and found that the 2 mm larger cancellous screws significantly increased the fixation strength within a synthetic osteoporotic bone model. Special care must be taken during implantation of larger than usual pedicle screws in bone of compromised quality since the weakened pedicles are more prone to fracture upon screw insertion [40, 43].

In addition to utilizing screws of a larger diameter, adjustments in the screw's length have been made to increase the depth achieved within the vertebral body in hopes of enhancing the pullout strength. It has been suggested that, in most cases, advancing the pedicle screw to about 80% of the vertebral body will provide sufficient fixation [33]; however, Zindrick et al. [32] noted that in osteoporotic specimens, there did not appear to be much of a difference between inserting the screw 50% into the vertebral body or complete insertion, without penetrating the anterior cortex. In these tests, the differences ranged from a 4% decrease to a 16% increase in pullout strength when fully inserted.

### 2.2. Conical Screws

**Figure 2:**
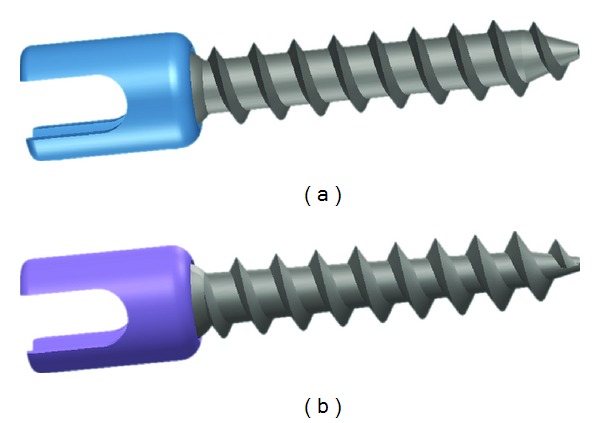
Pedicle screw designs. (a) Cylindrical threading and cylindrical core and (b) cylindrical threading and conical core.

Much of the research being done on pedicle screws involves those with a cylindrical shape (Figure 2(a)) [11, 15, 18, 35, 41, 43, 51, 53]. However, some have attempted to increase screw fixation by utilizing conical screws, which involve a tapering of at least the core of the screw to allow for a gradual increase in diameter in the proximal direction (Figure 2(b)) [17, 30, 38, 39]. The larger proximal diameter could lead to a decrease in fixation strength since a significant amount of stress is concentrated in the posterior portion of the screw, especially when screw turnback is necessary [17], but a conical shape could enhance pullout strength since the geometry could promote additional compression in the bone surrounding the periphery of the screw [30, 34, 54–57]. Theoretically, increased localized bone density could increase the screw's purchase within the vertebra. Furthermore, from an anatomical standpoint, the conical shape of the screw conveniently matches that of the pedicle, which has an elliptically shaped cross section [17, 18, 32, 53, 57] and anteriorly decreasing diameter [58]. The complimentary geometries of the screw and bone is beneficial since roughly 60% of the pullout strength of a pedicle screw is dependent on the cortical bone of the pedicle itself, while only 15% to 20% depends on the trabecular bone of the vertebral body [33, 59].

The effectiveness of conical pedicle screws, in comparison to standard cylindrical screws, in osteoporotic specimens has been controversial. Hsu et al. [30] noticed a significant increase in pullout strength between two models of conical screws (Cotrel-Dubousset and Texas Scottish Rite Hospital) as a result of compaction of the surrounding bone than with that of a cylindrical screw (the Moss Miami). However, a follow-up study [39] that compared a number of conical screws, containing different tapering patterns with cylindrical screws of similar diameters in an osteoporotic model, could not state any significant difference between any of the conical or cylindrical screws. Moreover, Chen et al. [17] performed a number of pullout tests comparing both types of screws in the presence and absence of bone cement in osteoporotic synthetic bone. For every augmentation technique that was performed, including the lack thereof, they determined that no statistical differences in pullout forces existed through the use of conical over cylindrical screws. On the other hand, Kim et al. [38] compared true cylindrical pedicle screws with those of a conical core and cylindrical thread as well as those with both a conical core and conical thread, finding that the conical core and cylindrical thread screws performed, on average, 23–37% better in pullout testing than true cylindrical screws and 10–21% better than the screws with a conical thread and conical core.

### 2.3. Thread Type

Developing a screw with proper thread design is essential in achieving optimal results within the human body as the preferred size, shape, and pitch will vary based on particular anatomy. For instance, in traditional mechanical design, a screw with a deep thread and large pitch is preferred in softer mediums to prevent stripping, while a smaller thread size and pitch are ideal where material strength may not be a concern, but size may be a limiting factor [50]. The osteoporotic spine, however, suffers from both a decrease in material properties [10] that would require a large screw with deep threads and from the aforementioned risk of pedicular fracture [40, 43], which limits the size of the screw that can be utilized.

From a purely mechanical perspective, the allowable load placed on a screw is dependent on the amount of surrounding material that contacts the thread [50]. Therefore, it could be hypothesized that by increasing the contact area between a pedicle screw thread and the surrounding bone, there will be a greater distribution of forces and thus larger pullout strength will be obtained. Krenn et al. [34] defined this coverage between thread and bone as the flank overlap area (FOA) and tested screws of varying thread types, pitches, and screw shapes to determine if this would be a good predictor of its fixation capabilities in bone of poor quality. Three screw designs were tested: one with a constant core diameter and V-threading (FOA = 206 mm^2^); another with a buttress thread and a conical core (FOA = 261 mm^2^); and the third with a varying thread design, conical core, and smaller pitch (FOA = 326 mm^2^). It was found that while the FOA was largest on the screw with the smallest pitch its pullout strength suffered since the bone between threads behaved more like bone fragments rather than compressed bone. On the other hand, the screw with the buttress thread and conical core outperformed the other two designs in whatever density the pullout test was performed in. Therefore, it was determined that bone compression achieved by having both a conical core and constant thread diameter, as well as an appropriate distance between threading, provided a better connection by means of friction than simply increasing the contact area between thread and bone.

Other attempts to increase fixation based on alterations of thread design could theoretically be achieved by increasing the number of threads being used. Based on the observation that a screw with two different threads on its polar ends has the capability to significantly compress nearby bone when used elsewhere in the body [60, 61], Mummaneni et al. [35] has suggested that adding a second parallel thread of smaller height to a pedicle screw would enhance the holding strength of the screw within elderly osteoporotic patients. After performing pullout tests between standard pedicle screws and the double threaded ones described above, it was determined that there was no significant difference between the two types and therefore the use of an additional thread in hopes of better compression to the surrounding bone would not be beneficial for use in osteoporotic individuals.

Also hoping to increase the fixation of pedicle screws through the addition of more than one thread, Brasiliense et al. [37] compared a standard single threaded screw with a pitch of 2.6 mm to a screw with a pitch of 4 mm and the addition of a second parallel thread in the proximal half. They hypothesized that while the standard screw had a larger overall FOA, the dual-threaded screw had a larger FOA in the region of the pedicle, which the majority of the pullout force is dependent upon [33, 59], and therefore should increase fixation within the bone. However, the dual-threaded screw achieved a pullout force 19% less than that of the single threaded screw when being tested in high porosity (low density) polyurethane foam models and performed only slightly better (7.8%) when tested in vertebrae with a bone density of less than 0.8 g/cm^2^. Both differences were determined to be statistically insignificant [37]. These results differ from those reported by Thompson et al. [36], who stated that the surface area of the screw's thread was a good predictor of its fixation strength within bone.

**Figure 3:**
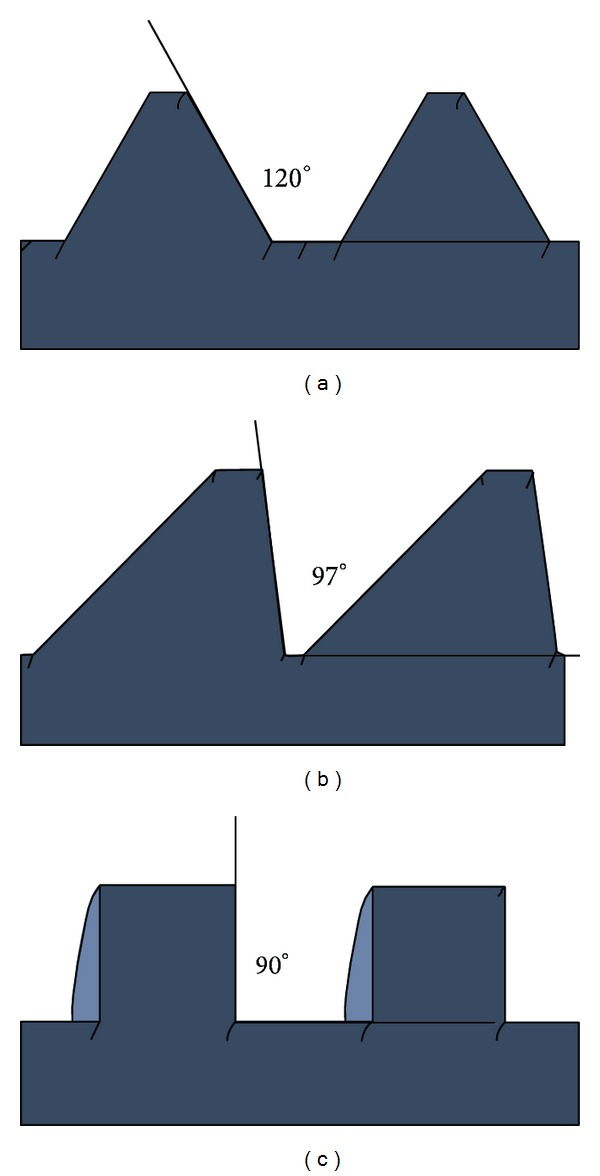
Posterior angle of various thread designs of pedicle screws: (a) standard, (b) buttress, and (c) square.

In addition to affecting the surface area making contact between screw and bone, the cross-sectional shape of the threading plays an important role in the function of the screw. Standard screw threads, closely resembling V-threads, named after the shape made by their cross section, are the most popular for use as fasteners (Figure 3). Other popular thread shapes include buttress and square threads, although these are used more as power screws for machine usage (nonmedical) as they are ideal for converting rotational motion to linear motion (Figure 3) [38, 50]. However, one potential advantage that these thread shapes have over the standard V-thread is that the thread height is made up of a near 90° angle to the axis of the screw as opposed to the standard screw's 120° (Figure 3). Comparing the above thread types to one another, Kim et al. [38] found that despite the geometric makeup of the threads, pullout forces of cylindrical screws with a V-thread were 16.3% and 13.4% greater than either the square or buttress threaded screws, respectively, when being tested in an osteoporotic model.

### 2.4. Material

In addition to altering the anatomical features of the pedicle screw, the screw material could also affect how well it is able to achieve proper anchorage in low quality bone. For instance, many pedicle screws are made out of stainless steel due to its biocompatibility and high strength [10, 13, 16, 28, 51, 62–66]; however, titanium has been considered to have superior mechanical and biological properties over stainless steel [51, 67]. For instance, with a lower modulus of elasticity, it is more flexible than stainless steel, which would allow for a reduction in stress shielding [12, 51]. Secondly, while it is considered to be a biocompatible material, it is also classified as bioactive which will thus promote osteointegration between the bone and screw [12, 51]. Finally, titanium is a material that allows greater magnetic resonance imaging (MRI) and computed tomography (CT) resolution over stainless steel [51].

To test if these characteristics resulted in an increase in anchorage of the pedicle screw within the osteoporotic spine, Christensen et al. [51] performed an *in vivo* study involving the use of 316L stainless steel and Ti-6Al-4V titanium pedicle screws in miniature pigs. After three months, the animals were sacrificed and then prepared for pullout testing to observe the effect the materials have on pullout strength over time. Despite the fact that there was noticeably better integration between the bone and titanium screws than that of the stainless steel screws, there was still a statistically insignificant increase of less than 5% in pullout strength of the titanium screw over the stainless steel.

In 2012, Shi et al. [12] performed mechanical testing on an expandable pedicle screw made out of a new titanium alloy, Ti-24Nb-4Zr-7.9Sn, in osteoporotic sheep. They hypothesized that this new alloy, which has an elastic modulus (42 GPa) closer to that of bone (~13.5 GPa) [68], would produce greater pullout forces than those made of Ti-6Al-4V (110–114 GPa) [12, 39]. After sacrificing the sheep six months postscrew implantation, it was noticed that there was, in fact, more bone surrounding the lower elastic modulus expandable screws than the higher modulus expandable screws. Following mechanical testing, it became apparent that this provided enough strength to significantly increase the pullout force of the low elastic modulus screws by 19.3% [12].

## 3. Insertion Technique

Before consideration of any type of augmentation is to be explored, it should first be determined if proper fixation can be achieved by varying the insertion technique used when implanting a pedicle screw. For instance, the pilot hole size is a very important variable to consider. An “oversized” pilot hole (bigger than the diameter of the screw being implanted) would prevent the screw from achieving adequate purchase within the bone, while an “undersized” hole (considerably smaller than the screw being used) would increase the installation torque required for screw implantation and, particularly in osteoporotic bone, this may increase the risk of fracture resulting in screw failure [62]. Other techniques that must be taken into consideration include the option of self-tapping screws versus pretapping pilot holes [9, 36, 62, 65, 69–71], screw angulation [14, 31, 72], screw depth [32, 62, 73], and, if necessary, screw turnback [17, 74].

### 3.1. Pilot Hole Size

As previously stated, the size of the pilot hole produced prior to screw insertion plays a very important role in pedicle screw fixation in osteoporotic bone. Battula et al. [62] set out to determine what they called the critical pilot hole size, which was defined as the hole size needed to maintain an optimal balance between low installation torque and high pullout strength. By performing pullout tests on pilot holes with diameters 70%, 71.5%, 73%, and 80% of the outer diameter of the screw being used, they determined that utilizing a pilot hole 71.5% of the outer diameter of the pedicle screw would satisfy this definition of critical pilot hole size.

### 3.2. Pretapped Hole versus Self-Tapping Screw

A topic of great interest is whether or not to utilize self-tapping screws or to rather have the pilot hole pretapped before screw insertion [9, 36, 69–71]. Self-tapping screws are often used in surgical applications as their ability to cut the thread path as it is being inserted greatly simplifies the procedure and, as a result, shortens the required time to perform the operation. Unfortunately, the screw will meet frictional resistance as it progresses, which will in turn increase both the installation torque and risk of fracture in low density bone [62].

The practice of pretapping a pilot hole was necessary as a means of accurately installing screws in longer bones in orthopedic surgeries, which was then implemented to spinal surgery [71], despite the controversy regarding its effectiveness. Chen et al. [9] determined that tapping the pilot hole prior to inserting the screw tends to weaken the pullout force and produce less consistent results than by utilizing self-tapping screws. Similarly, Pfeiffer and Abernathie [71] found that when testing ten different screw designs, each of which were either self-tapping or not, the majority of the self-tapping screws designs presented significantly stronger pullout forces in osteoporotic models. Alternatively, Carmouche et al. [69] reported that while the pullout force was lesser in pretapped holes in osteoporotic lumbar spine, there were no noticeable differences between the two techniques in the thoracic vertebrae. Furthermore, Thompson et al. [36] and Mehta et al. [70] were unable to find any noticeable differences in pullout strengths between tapped and untapped pilot holes.

While mixed observations are present regarding pretapping pilot holes, Helgeson et al. [75] hypothesized that an optimal insertional torque (IT) during the tapping process exists that would help predict the ideal screw size to be used in osteoporotic patients. During the pilot study, they calculated this optimal torque to be roughly 2.5 in-lbs. In each vertebra tested, one pedicle was tapped with increasing diameters until the IT reached 2.5 in-lbs and the tapping IT in the contralateral pedicle reached 1.5 in-lbs. Once this value was reached, the screw diameter to be used was determined to be the most recent tapping diameter plus 1 mm. Utilizing this technique, they observed a significant increase in pullout force of 23% in the pedicle that was tapped with 2.5 in-lbs IT as opposed to the one that had an IT of 1.5 in-lbs. Since no breaching of the pedicular wall occurred, these results can likely be explained by the larger diameter screws that were able to be used in the pilot holes tapped with an IT of 2.5 in-lbs.

### 3.3. Insertion Angle

Another possible technique for enhancing screw fixation is altering the angle through which it is inserted [14, 31, 72]. Patel et al. [31] performed tests on varying bone screws in synthetic models representing healthy (BMD = 0.32 g/cm^3^), osteoporotic (BMD = 0.16 g/cm^3^), and severely osteoporotic (BMD = 0.09 g/cm^2^) cancellous bone at angles ranging from 0° to 40°. They observed that while screws in healthy bone performed best at or near the angle of axial pullout, those in osteoporotic bone achieved the highest possible pullout strength around 10° to the axial force, while severely osteoporotic bone required screws to be positioned at a 40° angle to maximize fixation strength. The explanation behind these findings is likely because the purchase within the bone-screw interface is so poor in osteoporosis that maximizing the fixation would require an increase in bone concentration around the screw, as is achieved when the screw is pulled out at an angle from which it is inserted.

During instrumentation of a pedicle screw into a given vertebra, two common trajectories exist: the straightforward (0° to 10° both in the medial and caudal directions) and the anatomic trajectories (0° to 10° medial and 22° cephalocaudal) [14, 72]. Lehman Jr. et al. [76] showed that between the two trajectories the straightforward technique produces pullout forces 27% greater than those inserted using the anatomic trajectory in an osteoporotic vertebra. While both of these have the pedicle screw simultaneously engaged in cortical and trabecular bone, a third, lesser used trajectory (known as the “cortical bone trajectory”) keeps the screw completely engaged with the cortical bone of the pedicle [14]. Santoni et al. [14] performed pullout testing to compare this third method along with that of the anatomic trajectory. Despite the fact that the pedicle screws in the cortical bone trajectory were significantly smaller to avoid bicortical purchase (mean 29 mm versus 51 mm), it produced a mean pullout force of 367.5 N, while the anatomic trajectory produced pullout forces of 287.6 N.

### 3.4. Bicortical Fixation

If poor fixation is a concern, surgeons will often obtain additional strength by inserting the pedicle screw through the vertebral body and into the anterior cortex [29, 32, 49]. Breeze et al. [49] noted an increase in pullout strength of 26% to 44% between bicortical and unicortical screws, depending on the severity of osteoporosis. Zindrick et al. [32] demonstrated that, depending on the screw used, insertion through the anterior cortex resulted in an increase in pullout strength of 31% to 120% when compared to inserting the screw just up to the anterior cortex without penetrating it. Furthermore, Zhuang et al. [29] found that bicortical fixation within the sacrum can provide a greater resistance to pullout in the early stages of osteoporosis when being compared to a unicortical screw augmented with bone cement. However, as the level of osteoporosis worsens, this no longer remains the case.

When inserting a pedicle screw through the anterior cortex, special care must be taken. Due to the location of the aorta and iliac vessels along the anterior of the spine, incorrect placement of the screw could result in severe vascular injury [33, 40]. Additionally, Zindrick et al. [32] noticed during their testing that applying a cyclic load to the screws when attached to the anterior cortex resulted in a “windshield wiper” type motion as a result of the center of rotation shifting to the distal tip of the screw. Because of this, it was noted that there was an increased risk of pedicle fracture or screw bending. Therefore, it has been suggested that bicortical fixation should be reserved for use in the sacrum or in cases where the additional fixation achieved is desperately needed [33].

### 3.5. Hubbing

As pedicle screws are subjected to slight motions that occur as a result of forces from everyday activity, they undergo a “teeter-totter” effect with the pedicle acting as a fulcrum and the surrounding trabecular bone within the vertebral body being pushed away from the screw [73]. Paik et al. [73] postulated that this problem could be solved by “hubbing” the head of the pedicle screw, or placing it directly against the outer cortex of the pedicle. This would result in a smaller moment arm meaning that, when the same forces are placed on the screw, the resulting motions should be lessened; hence the severity of trabecular ablation is reduced. Unfortunately, following cyclic loading, it was noted that the pullout strengths for the screws utilizing the hubbing technique were significantly lower than those screws that were inserted with the head about 5 mm away from the cortex. It was speculated that this decrease may be a result of fractures that occurred during instrumentation; however, it was also noted that there were few differences between the pullout forces of hubbed screws in fractured pedicles and those where fracture did not occur. Furthermore, while there were no fractures observed when the screw was inserted normally, half of the pedicles that were implanted with the hubbing technique fractured during instrumentation. Therefore, it was concluded that hubbing a pedicle screw against the dorsal laminar cortex was not an adequate technique to enhance pedicle screw fixation.

### 3.6. Screw Turnback

While it is common during clinical instrumentation for the surgeon to perform necessary adjustments on the pedicle screw, it has been questioned as to whether or not this type of practice, such as in the case of turnback for screws that are inserted too far, would adversely affect screw fixation [17, 74]. Chen et al. [17] tested the effect of turning back pedicle screws 360 degrees in a low density bone model, representing severe osteoporosis, when the screw was inserted alone and when it was augmented with bone cement. They found that when adjusting the screw depth after a maximum of four minutes (prior to cement hardening), there was no noticeable difference in pullout strength from the screws that were initially inserted without backing out. Ying et al. [74] performed similar tests with bone cement augmentation in low density models but tested for both adjustments involving screwing out and screwing in a complete rotation after allowing the cement to fully harden. It was noticed that further advancement of the screw into the solidified cement proved to be detrimental to the bone-cement interface and thus had a significant negative impact on pullout strength. Turning the screw out a complete rotation also weakened the pullout strength but to a lesser extent since the screw was turning out of the cement and leaving the bone-cement interface largely unaffected. It can be gathered from these studies [17, 74] that minor adjustments to screw positioning during initial surgery, regardless of the addition or absence of bone cement, will have no major effect on screw fixation. However, after initial surgery, adjusting the depth of a screw augmented with cement should be avoided unless absolutely necessary.

## 4. Bone Cement

The strength of the trabecular bone in the vertebral body is dramatically diminished as osteoporosis progresses [10, 17, 24, 46]. As such, the bone-screw interface within the vertebral body becomes so poor that adjustments to the screw's design alone prove to be ineffective as conditions become more severe. Therefore, in some extreme cases, the addition of bone cements has been explored to enhance the screw's fixation within the vertebral body [9, 11, 13, 15, 16, 20, 21, 25, 28, 32, 41, 77]. Typically used in kyphoplasty or vertebroplasty procedures for restoring height in the vertebral body following compression fractures [16, 78, 79], introducing cement to strengthen the interface between the screw threads and its immediate surroundings has proven to be a successful, albeit controversial, solution for providing increased screw stability in bone of compromised quality [18, 20–22, 25, 26, 28, 29, 77].

### 4.1. Types of Cement

#### 4.1.1. Polymethylmethacrylate (PMMA)

Quite possibly the most frequently used cement, PMMA is also the most highly debated for its use in clinical practice [18, 20–22, 28, 29]. Less serious problems associated with the use of PMMA include its inability to be seen by common medical imaging techniques, such as X-ray. This, however, can be remedied by adding small amounts of barium sulfate to the mixture [24, 80]. More severe, however, is the polymerization of PMMA via an exothermic reaction [80–82]. Therefore, as it solidifies in the body, the cement will increase its temperature to about 40°C to 110°C [82] with one study reporting a temperature as high as 113°C [81], well within the range to allow thermal necrosis to take place to surrounding osteoblasts and neural tissue such as the spinal cord [81, 83]. Additionally, its injection into the vertebral body during its liquid phase presents a very real potential for leakage, further increasing the risk of neural injury [9, 11, 15, 17, 18].

Its inability to degrade under biological conditions poses even further threats. Since PMMA will remain in the vertebra as a permanent foreign body, if pedicle screw removal is desired, drastic, potentially damaging surgery will be required on the vertebra [13, 27, 84]. Furthermore, while PMMA may be a biocompatible material, its monomer methylmethacrylate (MMA) is in fact known to be toxic. It is believed that long term exposure to PMMA can result in MMA being absorbed into the blood stream resulting in cardiac issues such as embolic events [45] and hypotension [85].

Despite these concerns, the benefit that PMMA provides to screw stability in osteoporotic patients is often believed to outweigh the potential risks. In addition to its low cost and high availability [21], PMMA provides a mechanical strength like few other bone cements can. The literature has shown PMMA increasing the pullout strength of pedicle screws in osteoporotic vertebrae from 25% to 348%, depending on the amount used and technique of injection [11, 15, 16, 18, 20, 21].

Chen et al. [17] compared the pullout forces achieved in using conical screws alone in an osteoporotic synthetic model with standard cylindrical screws augmented with PMMA under the same conditions. While the conical screws reached a mean pullout force of only 35 N, the cylindrical screws with cement augmentation achieved forces averaging 298 N and 421 N, depending on how the cement was placed in the spine. Similarly, Liu et al. [21] found that standard pedicle screws augmented with PMMA attained average pullout forces 257% of those seen in pedicle screws without the cement but containing an expansive distal end.

#### 4.1.2. Calcium Phosphate (CaP) Cement

There are alternative bone cement options to use in the vertebral body when PMMA may be considered too hazardous of a material. One particular cement recently increasing in popularity is calcium phosphate (CaP) [13, 24–26]. Unlike PMMA, CaP is a biodegradable material. Consequently, after surgery is performed and as spinal fusion occurs, the cement will gradually degrade and allow newly formed trabeculae to take its place [13, 24, 86]. Additionally, CaP hardens via a hydration reaction, thus resulting in an endothermic response [13, 84]. Therefore, there is no heat produced as a result of the cement's usage and thus no risk of thermal necrosis to the surrounding tissue. There is, however, still the potential of leakage upon insertion which can, like PMMA, cause damage to the spinal cord [26]. Nevertheless, under careful placement by a skilled surgeon, the use of CaP can prove to be a safer option than PMMA when bone cement is desired.

One important drawback of using CaP cement is the weaker pullout force it induces when compared to that achieved by PMMA [23, 24]. Regardless, Yazu et al. [26] reported a rate of increase in pullout strength of about 244% when a cannulated pedicle screw augmented with CaP cement was compared with a standard nonaugmented screw in osteoporotic specimens. Furthermore, Stadelmann et al. [25] calculated that for every millimeter that CaP cement is added along the exterior of a cortically anchored pedicle screw, pullout strength will be increased by roughly 23 N (in other words, a pedicle screw with 15 mm of its shaft surrounded by CaP will increase its pullout strength by about 345 N).

As stated, a major advantage of using CaP over PMMA when choosing to augment a pedicle screw with bone cement is the fact that CaP will gradually degrade as new cancellous bone takes its place. Taniwaki et al. [13] performed a four-week study using beagles with induced osteoporosis to investigate how the use of CaP would affect the anchorage of pedicle screws over time as opposed to those being used without the bone cement. The dogs were sacrificed at one, two, and four weeks after surgery to perform a pullout test. It was noticed that, while there was an insignificant increase in pullout strength in the osteoporotic non-CaP group at four weeks compared to one week, there was a much more significant increase of 38.1% in the CaP treated osteoporotic dogs at week four compared to one week after surgery. Additionally, CaP increased the overall stability of pedicle screws in osteoporotic dogs as opposed to those without CaP by 28.1% to 56.3% from one to four weeks, respectively.

While CaP cement has been shown to provide a significant increase in pullout strength over the nonaugmented pedicle screw with less harmful side effects than PMMA, some may still be cautious of its use due to the ever present possibility of leakage outside the vertebral body. As a result, those surgeons who want to take advantage of the biocompatible characteristics of CaP while avoiding the potential hazards that could occur as a result of leakage may pack the pedicle screw's pilot hole with granular CaP particulate prior to screw insertion [77]. Knowing the advantages that it possesses, Hashemi et al. [77] performed pullout tests on screws augmented with CaP particles to see if it was still able to provide an increase in pullout resistance as opposed to nonaugmented screws. Using polyurethane blocks, it was determined that CaP particulates can increase the pullout strength in low density samples and can still be used to provide some additional strength in a rescue situation where the screw pullout already occurred and needs to be replaced. On the other hand, it was noticed that the augmentation technique tended to have an adverse effect on pullout strength in high density samples, indicating that it would not be ideal to use it in normal, healthy bone.

While the nature of the test performed by Hashemi et al. was meant to determine the mechanical benefits of augmenting screws with granular CaP particles immediately following surgery, it was suggested that further investigation needed to be performed in an *in vivo *environment to fully understand the potential of particulate CaP augmentation in a clinical setting. Since the experiment was performed in polyurethane blocks, there was a natural inability to test for how the potential of bone growth over time would affect the screw's pullout strength. An *in vivo* study would take into account the biomechanical effects that osteogenesis and cyclic loading has over time that cannot be properly tested *in vitro* [77].

#### 4.1.3. Other Biocompatible Bone Cements

A novel and quickly degradable cement made up of both calcium phosphate and calcium sulfate components was tested by Gao et al. [41]. They showed that using 2 mL of the cement to augment a standard pedicle screw in osteoporotic bone was enough to increase the pullout strength by 12.6%. While this was not considered to be a statistically significant increase, it was enough to produce comparable results to that of unaugmented screws in slightly denser osteopenic vertebrae. However, the cement did little to improve the level of fixation in severely osteoporotic specimens. A standard pedicle screw augmented with the calcium based cement only produced pullout strengths 6.2% greater than its unaugmented counterpart and 38.4% less than an unaugmented screw in osteoporotic bone.

Another popular biocompatible material used for enhancing the pedicle screw purchase is hydroxyapatite (HA). Similar to the calcium cements, HA is capable of promoting osteointegration between bone and screw. Hasegawa et al. [19] performed mechanical tests after sacrificing osteoporotic dogs who had HA coated screws implanted for ten days. Compared to the noncoated screws in each contralateral pedicle, HA increased the screw pullout strength by 60.6%. Following inspection of the screws, it was noted that there was significantly more bone growing between the threads of the HA coated screws as opposed to standard pedicle screws alone.

Similar to CaP, a bioactive cement consisting of strontium and hydroxyapatite nanoparticles (Sr-HA) that maintains a low curing temperature while promoting the formation of new bone over time has been evaluated [28]. Unfortunately, Zhu et al. [28] showed that under cyclic loading, PMMA outperformed Sr-HA cement by producing pullout forces nearly 50% stronger. Somewhat promising, however, they noticed that upon insertion of the pedicle screw, Sr-HA cement covered 79% of the length of the screw, while PMMA only covered 43%. This was suggested to be a result of Sr-HA's longer handling time and may produce more significant bone growth long term.

Calcium triglyceride (CTG) is biocompatible cement that benefits from a release of carbon dioxide during its early stages of polymerization. This forms pores within the material which in turn results in its expansion. While this increases concerns over cement extrusion, it is also believed that it could benefit screw fixation [20]. Hickerson et al. [20] found that, while PMMA increased pullout strengths by 25% over unaugmented pedicle screws, CTG increased these forces by 89%. Furthermore, when directly compared in revision situations, CTG augmented pedicle screws had pullout forces 30% larger than those augmented with PMMA.

### 4.2. Injection Techniques

**Figure 4:**
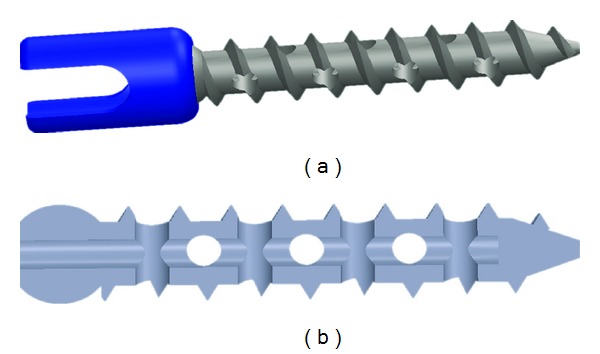
Fenestrated screws used for injection of cement after installation. (a) Model view and (b) section view (no head).

Multiple variables need to be considered when augmenting pedicle screws with bone cement in order to obtain both maximum fixation and safety. For example, it must be determined what the preferred method of applying the cement should be. One of the most popular is solid screw vertebroplasty, being the insertion of the cement into the pilot hole prior to placement of the screw [13, 15–17, 21, 22, 41, 63]. However, there are concerns over leakage occurring as the screw displaces the cement upon instrumentation [13, 17]. This led to a vertebroplasty technique involving fenestrated screws as a means of injecting the cement after the screw was already inserted (Figure 4) [9, 11, 15, 17, 18, 63]. Chao et al. [87] showed that, while statistically insignificant, prefilling the pilot hole with cement produces pullout forces 40% greater than fenestrated injection. Both prefilling and injection techniques, however, were significantly stronger (461.7% and 301.5%, resp.) than unaugmented pedicle screws in osteoporotic vertebrae.

A third injection technique exists called balloon kyphoplasty. This technique involves a medical balloon placed into the vertebral body and then expanded to create a cavity in the trabecular bone. After the balloon is deflated and removed, cement is injected into the cavity followed by screw insertion [15, 16].

Becker et al. [15] determined that, of the three techniques discussed (vertebroplasty injection, vertebroplasty injection involving fenestrated screws, and balloon kyphoplasty), both vertebroplasty injections performed greater than the kyphoplasty technique from a mechanical standpoint, producing near identical results of just under an 80% increase in pullout strength over unaugmented screws. In a separate test, Burval et al. [16] found that kyphoplasty based cement injection provided more significant pullout forces than did the vertebroplasty technique of injecting the cement prior to screw insertion. However, the differences between these two studies may be a product of the variations in testing procedures. For instance, Burval et al. [16] compared the two augmentation techniques in the same specimen whereas Becker et al. [15] limited each specimen to one of the techniques being tested. Furthermore, Burval et al. [16] used 4 mL of PMMA in the kyphoplasty augmentation and only 2.5 mL of the cement for vertebroplasty. Becker et al. [15], on the other hand, used 2 mL PMMA for all of the augmentation tests performed. They did admit, however, that a larger amount may be required for the kyphoplasty technique since using such a small amount may not be enough to properly incorporate itself into the surrounding bone after cavity formation in the vertebral body.

Becker et al. [15] also noted that, of the ten specimens that were tested via cement injection through the cannulated screw, two resulted in leakage into the epidural veins. This is in direct contrast with how this technique was expected to perform when compared to solid screw vertebroplasty [13, 17]. Chen et al. [9] reaffirmed that there is a risk of cement extrusion outside of the vertebral body when being injected through a cannulated screw with proximally located radial holes near the posterior cortex of the vertebral body. However, they also noted that the pullout forces required to remove one of these screws augmented with PMMA is significantly larger than one where the cement is extruded from the distal portion of the screw. This suggests that upon careful insertion by a skilled surgeon in a vertebra not already prone to leakage (i.e., no identifiable breaches in any of the walls), the added strength achieved by injecting PMMA through a screw with radial holes located just past the pedicle-vertebral body junction may be worth the risk associated with it if fixation is particularly difficult to achieve.

### 4.3. Optimal Volume

Another important factor to consider when using bone cement to augment pedicle screws is the optimal volume to utilize. Paré et al. [11] conducted research on the optimal amount of PMMA, by testing pullout strengths of screws augmented with three different volumes: 0.5, 1.0, and 1.5 cc in the thoracic spine and 1.5, 2.0, and 2.5 cc in the lumbar spine. Perhaps somewhat surprising, their numbers suggested that more cement is not always better. Maximum pullout forces in the thoracic spine occurred with 1.0 cc of bone cement augmentation (186% over the nonaugmented control) and in the lumbar with 1.5 cc of cement (264% over the nonaugmented control). In both cases, as larger amounts of cement were added, there was a gradual decrease in the difference between its pullout strength and that of the control in the contralateral pedicle. Furthermore, when less cement was used in the thoracic spine, any mechanical advantage that was achieved over the control was so small that it was deemed statistically insignificant.

## 5. Novel Pedicle Screws

### 5.1. Expandable Screws

**Figure 5:**
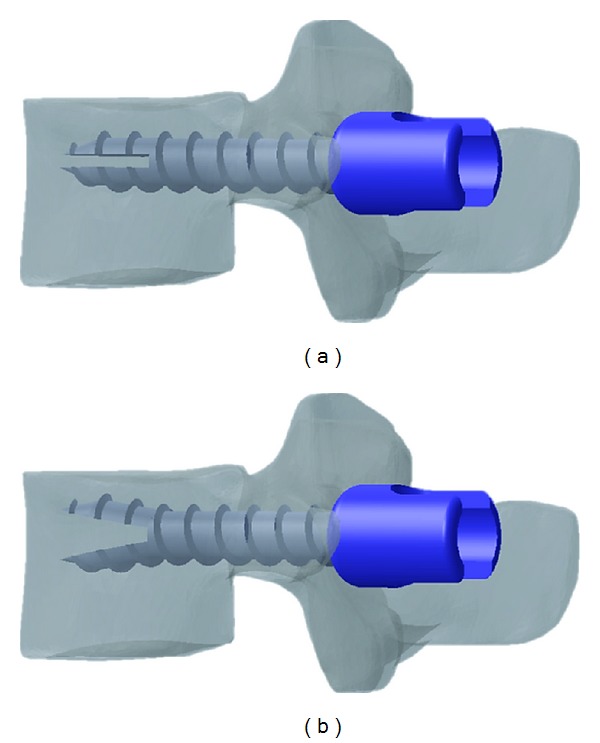
Expandable screw in the vertebral body (a) unexpanded and (b) expanded.

Since increasing a pedicle screw's diameter in an attempt to achieve better purchase will also increase the likelihood of pedicle fracture [40, 43], screws allowing their insertion into the pedicle and vertebral body in a similar fashion to that of standard cylindrical screws but expanding distally after their insertion were developed [12, 18, 21, 22, 40–44]. This increase in diameter at the screw's tip concentrates fixation in the vertebral body for better anchorage in the trabecular bone without compromising the integrity of the pedicle (Figure 5) [22, 40, 41, 43].

Cook et al. [18] compared the pullout strengths in severely osteoporotic specimens of a conventional pedicle screw (6.5 mm diameter) augmented with PMMA with an unaugmented expansive screw that consisted on four finned expandable screws expanding along the distal two-thirds of the screw (OMEGA-21, 7 mm diameter and 8.5 mm distal expansion). Mean pullout forces to the cement augmented screw were measured at 104.66 N. While the expansive screw alone measured at 81.12 N, this difference was not determined to be significant. This suggests that when safety is a concern, it may be a better alternative to use the expansive screw rather than taking the risks associated with cement injection.

Koller et al. [42] also tested their own version of a four finned expandable screw, but one where only the distal most one-fifth of the shaft length expanded. Expansion in this case resulted in an increase of pullout strength of roughly 20% when compared to standard screws of similar dimensions. This difference, however, fell just shy of being considered statistically significant.

Gao et al. [41] performed pullout tests on another design of distally expansive pedicle screw that contains two expanding fins (Figure 5) rather than four. The fins, once fully extended, increase the distal diameter of the screw by roughly 2.5 mm. By testing this screw against standard pedicle screws, evidence suggests that the expansion mechanism significantly increases pullout strength of 27.2% and 51.5% in osteoporotic and severely osteoporotic specimens, respectively. Additionally, the expansive pedicle screw alone produced pullout forces 42.7% greater than a standard screw augmented with calcium-based cement in severely osteoporotic bone.

While still larger, the differences were less noticeable between two finned expansive pedicle screws and PMMA augmented standard screws in pullout tests performed by Wu et al. [44]; the expansive pedicle screws alone produced 7.3% greater pullout forces than standard screws with 2 mL of the bone cement in osteoporotic vertebrae and only 3.3% greater in specimens with severe osteoporosis. However, in both levels of osteoporosis, it was recorded that an expansive pedicle screw augmented with PMMA provides greater pullout strength when compared to a conventional pedicle screw, both in the presence and absence of bone cement.

More recently, Liu et al. [21] tested a similarly designed two finned expansive pedicle screw (6.5 mm diameter and 7.5 to 8.5 mm distal expansion) to standard pedicle screws (6.5 mm diameter) both augmented with and without PMMA in low density synthetic bone. It was shown that the expansive pedicle screws had pullout strength 25.3% to 48.4% greater than any of the standard pedicle screws used. However, unlike the previous studies, it was determined that the pullout strength of the expansive pedicle screw was significantly lower than that of the cement augmented standard screw. They suggested that this likely has to do with the fact that the expansive screws experience a localized increase in diameter at the distal-most point only, while the PMMA augmented screw has an increased diameter uniformly throughout the entirety of the screw.

### 5.2. Expandable Anchors

While a mechanism allowing for distal expansion in pedicle screws has shown to provide promising results as a means of fixation within osteoporotic bone, there still exists concern as to whether trabecular bone of compromised quality is strong enough to take full advantage of the increased diameter generated [45]. Since only about 20% of the fixation strength of a pedicle screw occurs in the trabeculae of the vertebral body [33, 59] and, in cases of osteoporosis, the integrity of trabecular bone becomes compromised much faster than that of the cortical bone [10, 17, 24, 46], it may benefit pedicle screw fixation to choose a design that takes advantage of the additional strength that is left from the cortex. As a result, some have questioned if expanding the diameter of the screw immediately at the posterior cortex of the vertebral body, thus allowing an increased surface area to provide anchorage to the stronger cortical bone, would help in increasing the fixation strength of pedicle screws in osteoporotic individuals [10, 45].

Lin et al. [10] found that the addition of an external shell with expandable wings, similar to a wall anchor used in drywall, performed significantly better in L1 to L4 porcine segments where the vertebral body was hollowed out to simulate osteoporosis. Interestingly, they discovered that a point is reached where further expansion of the wings causes more harm to instrument fixation as the additional distance achieved by the stainless steel anchor actually placed more stress on the surrounding bone, thereby increasing the risk of fracture. In their current porcine model, however, they determined that the optimal wing height was achieved at 3.75 mm, which increased its pullout strength by 47% when compared to standard pedicle screws alone.

Vishnubhotla et al. [45] performed pullout testing on a 6.5 mm expansive pedicle screw (Alphatec Osseoscrew) that opens up to a maximum diameter of 10 mm just past the opening of the pedicle-vertebral body junction. The result of this was an increase of pullout strength of 29% when being compared to standard pedicle screws of otherwise similar dimensions.

A significant advantage this technique has over other proposed fixation enhancement techniques, such as PMMA augmentation, is its reversibility. While removal of a cement augmented screw may require some degree of vertebrectomy, the removal of expandable screws and anchors can be accomplished by simply retracting the device and then removing it as one would a standard pedicle screw.

## 6. Other Techniques

### 6.1. Triangulation

Pedicle screw coupling, or triangulation, involves the connection of screws inserted in the bilateral pedicles of a single vertebra via a rod or plate being placed between them [66, 88]. Rather than depending on the integrity of the bone-screw interface for an individual screw, the fixation will rely on the mass that exists between the two screws [88]. Ruland et al. [88] found that in cases of osteoporosis, coupling pedicle screws with a connecting plate will significantly increase the pullout strength in comparison to single screws or laminar hooks. Suzuki et al. [66] arrived at a similar conclusion between coupled and standalone pedicle screws, so long as they were not being used in cases of severe osteoporosis, which exhibited no statistical difference. Furthermore, they also determined that there is no improvement in pullout strength observed when using a double coupler system as opposed to a single coupler of adequate stiffness between pedicle screws.

### 6.2. Double Pedicle Screws

While the majority of pedicle screw pullout studies involve the use of a single screw being inserted in a pedicle [10, 14–16, 18, 28, 32, 45, 51, 72], it is possible to take advantage of the elliptical cross section of the pedicle [17, 18, 32, 53, 57], to create constructs with multiple screws in a single pedicle [53]. Jiang et al. [53] tested the effect that inserting two pedicle screws into a single, slightly larger than average, pedicle in the thoracolumbar spine would have on pullout strength. They hypothesized that two 5 mm diameter pedicle screws positioned at a 10° to 15° angle from each other within the same pedicle would perform better than a single 6 mm screw would alone. It was observed, however, that while there was a 7.5% increase in pullout strength this difference was determined to be insignificant. It was hypothesized that this result was likely due to the bulkier screw system compromising the strength of the osteoporotic pedicle. Because of this and the observation that there was interference between the two screws during instrumentation, it was suggested that 4 mm pedicle screws instead of 5 mm may provide the additional fixation desired while leaving the pedicle uncompromised.

### 6.3. Hooks

Often times, in cases where it may be particularly unsafe to utilize pedicle screws in a given vertebra, laminar hooks are used in its place. Popular for usage in the cervical and thoracic levels due to the small size of the pedicles, laminar hooks attach to the exterior cortical bone of the lamina [46, 47, 89, 90]. In cases of osteoporosis, this can be seen as a huge advantage over pedicle screws which rely on the strength of cancellous bone for much of its fixation despite the fact that the integrity of this bone is more severely compromised in earlier stages of osteoporosis than the cortical bone [10, 17, 24, 46]. The downside of this type of fixation, however, is that while screw failure will occur mostly by stripping of the surrounding bone [31, 32, 46, 51] failure of hooks tends to result more in catastrophic bone fracture [46]. Cordista et al. [46] compared, in thoracic levels of osteoporotic specimens, the stability of pedicle screws with a claw design, which was described as a superior laminar hook combined with an inferior pedicle hook. It was observed that the claw design required roughly an 88% increase in force to remove than the pedicle screw did. Additionally, it was noted that during disarticulation and instrumentation, eight of the sixty vertebra used were damaged prior to testing and six of these occurred in the T4 to T8 levels which contained the smallest pedicle sizes and lowest BMD.

Hackenberg et al. [47] likewise compared the pullout strength of pedicle screws with pedicle and laminar hooks in the thoracic spine, however, limited the instrumentation to one of the hooks per level (pedicle hooks from T4 to T8 and laminar hooks from T9 to T12). While pedicle screws outperformed hooks in healthy bone, there was no difference observed between the two techniques in specimens with low bone density. However, it was also noted that they, too, had difficulties inserting pedicle screws in the smaller pedicles of the T4 to T8 levels. This further reinforced the need to consider hooks as an alternative to pedicle screws in bone where risk of fracture is particularly high, especially at the end of constructs, where cantilever forces are greatest.

## 7. Conclusion

Obtaining adequate fixation of a pedicle screw in fragile bone, as that of an osteoporotic patient, has been proven to be an ongoing challenge. From a design standpoint, proper threading and material choices appear to be the most effective way of safely increasing the pullout strength of a standalone pedicle screw. A standard or V-shaped thread produces pullout forces 16.3% and 13.4% greater than either square or buttress threading, respectively, in standard cylindrical screws, with even more fixation possible when the threads are self-tapping. Additionally, the use of a biocompatible material with an elastic modulus closely resembling that of human bone can achieve high pullout forces through osteointegration and a reduction of stress shielding. In more challenging situations, however, the added stability of a larger diameter can be achieved with the acceptance of an increased risk of bone fracture. Similarly, longer screws are most effective to be used in bicortical fixation; but if performed above the sacrum, special care must be taken to avoid accidental penetration of the vessels.

During insertion, choosing a screw trajectory that involves a high amount of purchase in the cortical bone can help to enhance screw fixation. Both the straightforward and cortical bone trajectories produced pullout forces about 27% larger than those seen in the more traditional anatomic trajectory. Alternatively, pedicle screw coupling has been shown to increase the pullout strength of pedicle screws as opposed to those inserted alone, while the placement of two smaller screws in a single pedicle produced insignificant differences.

PMMA augmentation has been proven to increase pedicle screw pullout forces by up to 348% in some situations, but its high polymerization temperature, inability to degrade, and risk of leakage are among some of the hazards associated with its use. While more biodegradable cements such as CaP and HA generally do not increase pullout forces to the extent of PMMA, the benefits associated with them tend to make them the safer and more attractive option when cement usage is considered. Alternatively, it has been shown in some cases that expansive pedicle screws have a comparable pullout strength to standard screws augmented with PMMA and 42.7% greater forces than screws augmented with a calcium-based cement. Therefore, these screws prove to significantly increase pullout forces in low quality bone while remaining a safer option over the use of bone cement.
